# A Case Report on Netherton Syndrome

**DOI:** 10.7759/cureus.9166

**Published:** 2020-07-13

**Authors:** Wiel O Abdalrheem, Omar B Alluhayyan, Abdulmajeed Alharbi

**Affiliations:** 1 Dermatology, King Saud Hospital, Qassim, SAU; 2 Medicine, Qassim University, Qassim, SAU

**Keywords:** netherton syndrome, atopic diathesis, congenital ichthyosis, trichorrhexis invaginata

## Abstract

Netherton syndrome (NS) is a rare form of skin disorder characterized by extensive skin desquamation, hair shaft abnormality and atopic manifestations. We report a case of a two-year-old girl brought to our dermatology clinic by her mother, who had a generalized scaly skin lesion that started at birth. Her family history revealed a similar case in two of her sisters. A skin examination revealed diffuse serpiginous erythematous pruritic plaques, surrounded by double-edged scales beside her hair shaft defects. The patient was diagnosed with NS, and we began treatment using topical emollients, antibiotic and corticosteroid.

## Introduction

Netherton syndrome (NS) is a rare severe autosomal recessive disorder of ichthyosis characterized by extensive skin desquamation, hair shaft abnormality and atopic manifestations with high immunoglobulin E (IgE) levels and hypereosinophilia. The precise incidence of NS is unknown but is estimated to be present in 1:100,000 to 1:200,000 live births [1,2]. This syndrome presents at or soon after birth with the following characteristics: generalized erythroderma, scaling and/or continuous peeling of the skin that resembles sparse, brittle hair containing trichorrhexis invaginata. Generalized scaly erythroderma is apparent soon after birth and usually persists. Ichthyosis linearis circumflexa (ILC), which typically consists of serpiginous patches bordered by double-edged scales, develops during the first years of life in patients with NS. NS is attributed to mutations in the gene encoding the serine protease inhibitor Kazal-type 5 (SPINK5) [3,4]. We report a child Saudi female with this syndrome and briefly review the literature.

## Case presentation

**Figure 1:**
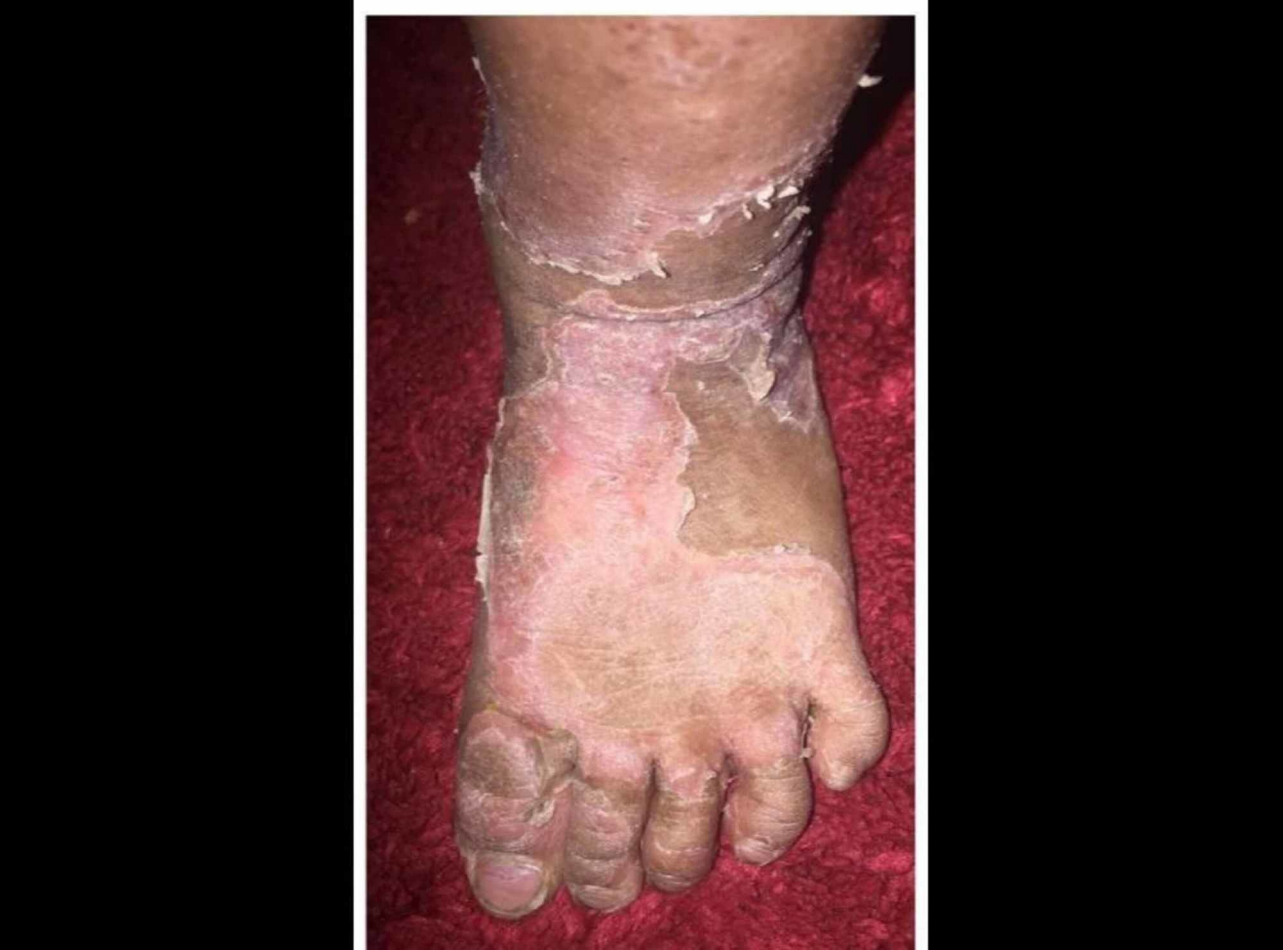
Ichthyosis linearis circumflexa (ILC) presents as serpiginous patches bordered by double-edged scales at the margins.

**Figure 2:**
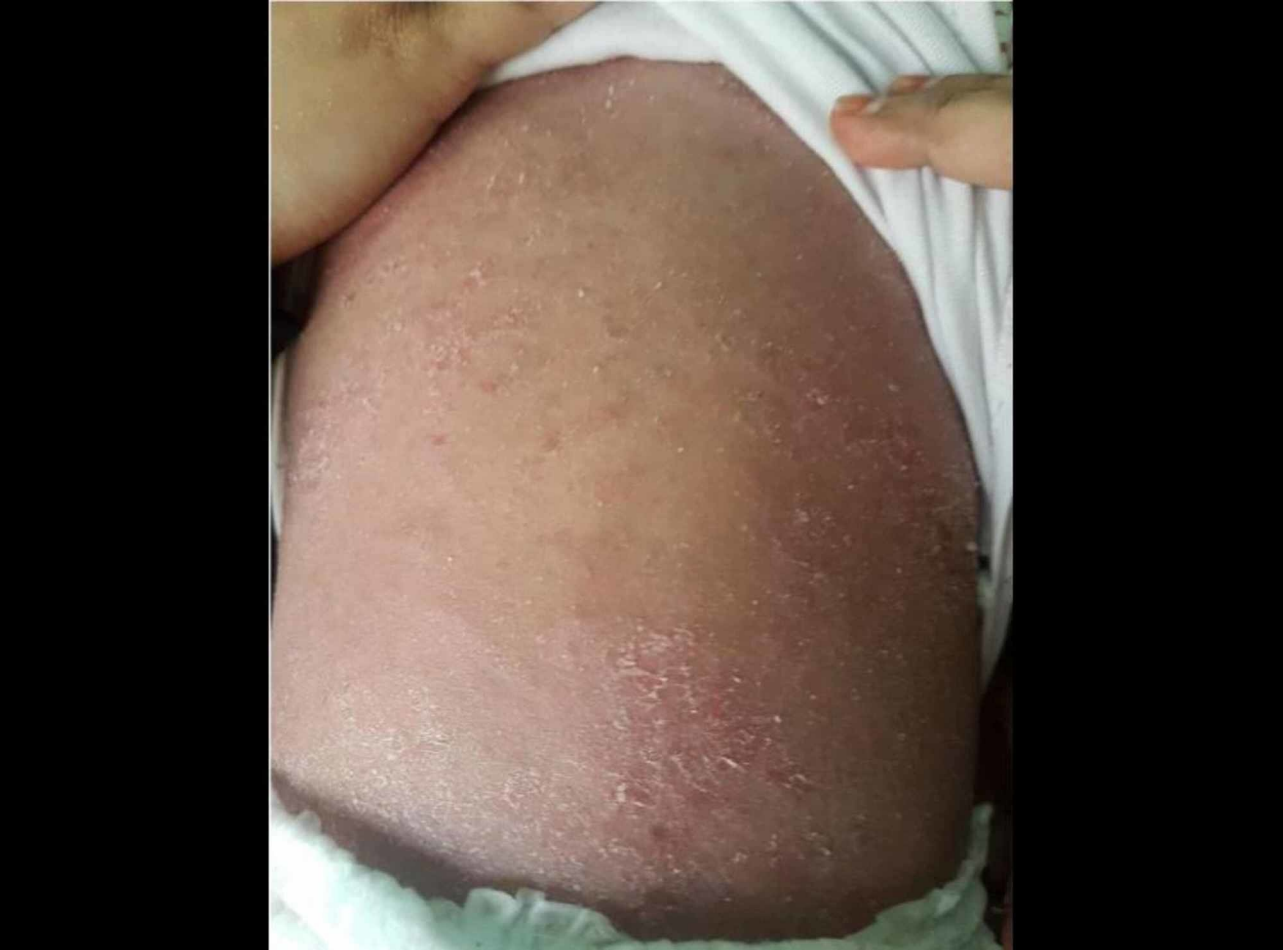
Diffuse erythematous plaques with white scale involving the abdomen and the back.

**Figure 3:**
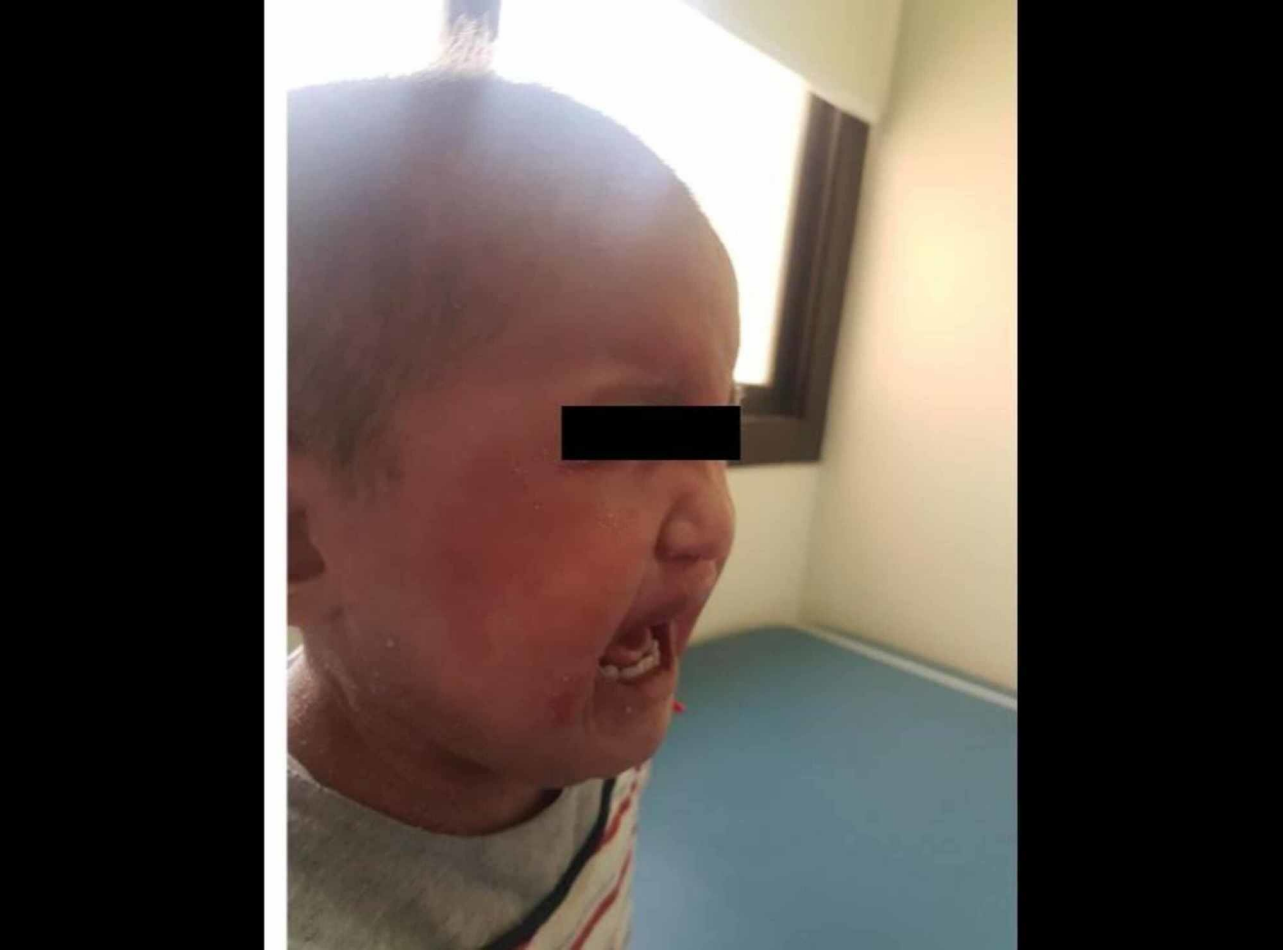
Short, thin, sparse and brittle hair on the scalp.

A two-year-old girl was brought by her mother to our dermatology clinic in King Saud Hospital, with the main concern of generalized scaly skin lesions since birth. The lesions started from birth when the mother noticed scaling over her daughter’s scalp. A few months after birth, these lesions progressed to involve the face, neck, abdomen, and upper and lower limbs. Later, the mother noticed a similar erythematous scaly lesion in the diaper area. The baby was experiencing a severe itch and presented with an offensive smell all over the affected areas of her body. The scaling of the skin was aggravated by dry, hot climate and partially improved with emollients. She was born at full-term without a collodion membrane, and had an uneventful pregnancy and delivery. Her two sisters had a similar problem since infancy and her parents were first-degree cousins. A physical examination revealed that there were widespread serpiginous erythematous pruritic plaques, surrounded by a double-edged scales characteristic of ILC in the extremities (Figure 1). Erythema and desquamation were also prominent on the abdomen (Figure 2). A dermatological examination of the scalp hair showed diffuse scaling as well as sparse, lusterless, and short hair (Figure 3).

The patient’s teeth, nails, eyes, and mucous membrane were normal. Her blood investigation revealed peripheral eosinophilia (absolute eosinophil count >500 cells/mcL). The infant received a diagnosis of NS according to the strict published Diagnostic criteria [5]. Cutaneous lesions were managed using topical medications such as moisturizers, antibiotic and corticosteroid. 

## Discussion

NS is a rare skin disorder that commonly begins at birth. It can present with multiple lesions and variable clinical manifestations, which may cause a delay or lack of diagnosis [6]. The main differential diagnosis of cutaneous manifestations in our patient includes atopic dermatitis, autosomal recessive congenital ichthyosis and peeling skin syndromes. However, proposed diagnostic criteria for NS requires the presence of any one of the following criteria items beside allergic manifestation: scaling erythroderma, specific hair shaft defect, history of NS in a sibling or identification of SPINK5 mutation [5]. Despite the unavailability of light microscopy and DNA sequencing analysis, the picture of our patient confirms the diagnosis of NS. Beyond the neonatal period, skin changes emerge into ichthyosis linearis circumflexa which are polycyclic migratory plaques with unique peripheral double-edged scaling [7]. The finding of trichorrhexis invaginata is specific for NS [3]. Manifestations of atopy may present as atopic dermatitis or asthma with elevated IgE [8]. NS usually carries favorable improvement with age, and patients evolve to a milder localized phenotype over time [9]. Treatment is not well defined, as various modalities have been prescribed. Topical agents have been used, such as corticosteroids, calcineurin inhibitors and retinoids. For severe cases, intravenous immunoglobulin and anti-tumor necrosis factor α (anti-TNF-α) are therapeutic options [10].

## Conclusions

Early recognition of unusual skin disorders, for example, NS, remains a challenge due to the difficulty of obtaining specialist guidance and lack of provider recognition. Dermatologists should be aware of the similarities between NS and other Inflammatory skin lesions. A prompt high index of suspicion is required for scaly erythroderma that is unresponsive to the usual treatment of atopic dermatitis.
